# Expression of RUNX1 Correlates with Poor Patient Prognosis in Triple Negative Breast Cancer

**DOI:** 10.1371/journal.pone.0100759

**Published:** 2014-06-26

**Authors:** Nicola Ferrari, Zahra M. A. Mohammed, Colin Nixon, Susan M. Mason, Elizabeth Mallon, Donald C. McMillan, Joanna S. Morris, Ewan R. Cameron, Joanne Edwards, Karen Blyth

**Affiliations:** 1 Transgenic Models Lab, Cancer Research UK Beatson Institute, Glasgow, Scotland, United Kingdom; 2 Academic Unit of Surgery, College of Medical, Veterinary and Life Sciences, University of Glasgow, Glasgow, Scotland, United Kingdom; 3 University Pathology Unit, Southern General Hospital, Glasgow, Scotland, United Kingdom; 4 School of Veterinary Medicine, University of Glasgow, Glasgow, Scotland, United Kingdom; 5 Institute of Cancer Sciences, University of Glasgow, Glasgow, Scotland, United Kingdom; University of Eastern Finland, Finland

## Abstract

The RUNX1 transcription factor is widely recognised for its tumour suppressor effects in leukaemia. Recently a putative link to breast cancer has started to emerge, however the function of RUNX1 in breast cancer is still unknown. To investigate if RUNX1 expression was important to clinical outcome in primary breast tumours a tissue microarray (TMA) containing biopsies from 483 patients with primary operable invasive ductal breast cancer was stained by **immunohistochemistry**. RUNX1 was associated with progesterone receptor (PR)-positive tumours (P<0.05), more tumour CD4+(P<0.05) and CD8+(P<0.01) T-lymphocytic infiltrate, increased tumour CD138+plasma cell (P<0.01) and more CD68+macrophage infiltrate (P<0.001). RUNX1 expression did not influence outcome of oestrogen receptor (ER)-positive or HER2-positive disease, however on univariate analysis a high RUNX1 protein was significantly associated with poorer cancer-specific survival in patients with ER-negative (P<0.05) and with triple negative (TN) invasive breast cancer (P<0.05). Furthermore, multivariate Cox regression analysis of cancer-specific survival showed a trend towards significance in ER-negative patients (P<0.1) and was significant in triple negative patients (P<0.05). Of relevance, triple negative breast cancer currently lacks good biomarkers and patients with this subtype do not benefit from the option of targeted therapy unlike patients with ER-positive or HER2-positive disease. Using multivariate analysis RUNX1 was identified as an independent prognostic marker in the triple negative subgroup. Overall, our study identifies RUNX1 as a new prognostic indicator correlating with poor prognosis specifically in the triple negative subtype of human breast cancer.

## Introduction

Breast cancer is the third most common cause of cancer death in the UK, accountable for more than 11,000 deaths in 2011 alone (www.cancerresearchuk.org) and an estimated 39,620 female deaths in the USA in 2013 (www.cancer.gov). In human breast cancer, oestrogen receptor (ER), progesterone receptor (PR), and human epidermal growth factor receptor 2 (HER2) are well-established prognostic and predictive markers, and testing for them is now considered standard of care [1]. Based on the receptor status, human breast cancer can be subdivided into three main groups: oestrogen receptor positive (ER+), human epidermal growth factor receptor 2 positive (HER2+) and triple negative (ER−/PR−/HER2–). ER+ and HER2+patients benefit from targeted treatments such as Tamoxifen and/or Trastuzumab which have consistently improved disease outcome [2]. On the other hand, the triple negative (TN) subtype lacks any specific targeted therapy and is associated with worse overall prognosis in comparison with the other subtypes [3]. This underlines the urgent need for new prognostic and therapeutic targets specific for this group of patients.

The *RUNX* genes are a family of three transcription factors (RUNX1, 2 and 3) known to play essential roles in haematopoiesis, osteogenesis and neurogenesis [4]. Besides being key developmental regulators, *RUNX* genes are also important in cancer, acting both as oncogenes or tumour suppressors in different systems [5]. *RUNX1* is the most frequently mutated gene in human leukaemia and many studies have focused on its tumour suppressive function in haematopoietic malignancies [6]. However, in recent years, a new role for RUNX1 outside the haematopoietic system has started to emerge with several studies indicating how this transcription factor could be more broadly implicated in cancer [7],[8]. In particular RUNX1 has been identified as a key regulator of tumourigenesis in various epithelial cancers [9]–[11]. However little is known about the role of RUNX1 in human breast cancer [12]. Wang and colleagues using 3D culture models showed that *RUNX1* deletion in MCF10A acini resulted in increased cell proliferation and abnormal morphogenesis [13]. In addition, three independent large scale sequencing studies on human breast cancers discovered recurrent *RUNX1* mutations and deletions in human tumours [14]–[16] while Kadota *et al* showed by qRT-PCR on a small breast cancer cohort (29 samples) that *RUNX1* downregulation is associated with high-grade primary breast tumours [17]. Here we have carried out the first comprehensive characterization of RUNX1 expression in tissues from a large cohort of human breast cancers and demonstrate its prognostic value in different tumour subtypes.

## Materials and Methods

### Patients

The expression studies in human tissues were ethically approved from West of Scotland Research Ethics Service West of Scotland REC4 (REC Ref: Project Number 02/SG007(10), R and D project: RN07PA001). Consent was not obtained, but all patient information is anonymised with all patient identifiers removed. Patients diagnosed with invasive breast cancer at three Glasgow hospitals (Royal Infirmary, Western Infirmary and Stobhill Hospital) between 1995 and 1998 were studied (n = 483). Clinical and pathological data including age, histological tumour type, grade, tumour size, lymph node status, lymphovascular invasion, type of surgery and use of adjuvant treatment (chemotherapy, hormonal therapy and radiotherapy) were retrieved from the patient records and histopathology reports.

### Tissue microarray (TMA) construction and immunohistochemistry

Tissue microarrays (TMA) were already available for use in this study. 0.6 mm^2^ cores of breast cancer tissue, identified by the pathologist (EM), were removed from representative areas of the tumour taken from breast cancer patients at the time of surgical resection. All tissue microarray blocks were constructed in triplicate and were utilized to assess ER, PR, HER2 status, Ki-67 and microvessel density by immunohistochemical analyses as previously described [18]–[21]. Immunohistochemistry was used to quantify cellular infiltrate of macrophages [19], CD4+, CD8+lymphocytes and CD138+plasma cells as previously reported [22].

#### Immunohistochemistry for RUNX1

RUNX1 antibody (Sigma, HPA004176) was validated to confirm its specificity by western blot (Figure S1). Expression was detected in a positive control (T6i) but not in a leukaemia cell line deleted for RUNX1 (3SS cells). Human mammary epithelial cells (hMEC) transfected with a RUNX1 overexpression vector (hMEC-*RUNX1*) or empty vector (hMEC-Puro) were used as an independent validation. TMAs were stained for RUNX1 by immunohistochemistry. Heat induced epitope retrieval for RUNX1 was performed at 98°C for 25 minutes in citrate buffer (pH 6). Endogenous peroxidase was blocked by incubation in 3% hydrogen peroxide (DAKO, UK) for 5 minutes. The cores were then incubated with primary antibody for RUNX1 added at dilution of 1∶100 for 40 minutes at 25°C. Sites of binding were detected using the appropriate Envision secondary antibody (DAKO code K4003) and visualized using DAB (3-3′ diaminobenzidine, DAKO, UK) according to the manufacturer’s instruction. Cores were counterstained with haematoxylin, dehydrated and coverslipped with DPX.

#### Weighted histoscore method

RUNX1 staining was quantified using the weighted histoscore method to give a value of 0–300 [23]. One hundred and fifty cores (10% of total core number) were scored independently for epithelial RUNX1 expression by two observers (NF and ZM) blind to patient’s outcome and the other observer’s score. Interclass Correlation Coefficient (ICC), measure of inter-observer agreement, was 0.82. NF then scored all cores and this data was used in subsequent analysis.

### Statistical analysis

Inter-relationships between variables were assessed using contingency tables with the chi-squared test for trend as appropriate. Univariate analysis and multivariate survival analysis with calculation of hazard ratios (HR) were performed using Cox’s proportional-hazards model. A stepwise backward procedure was used to derive a final model of the variables that had a significant independent relationship with survival. Mortality incidences up to March 2010 were included in the analysis. Analysis was performed using SPSS software version 19 (SPSS Inc., Chicago, IL, USA).

### Cell lines

T6i leukaemia cell line overexpressing RUNX1 [24] and 3SS (a cell line generated from a murine lymphoma which is genetically deleted for RUNX1 and kindly provided by Gillian Borland in ERC’s lab) were used respectively as positive and negative controls in RUNX1 western blots. The genetically altered mouse used to generate 3SS was covered by University of Glasgow ethical review process and project licence PPL60/4408. hMEC-TERT cell line [25] (a kind gift of Barbara Chaneton) was grown in HuMEC complete media (Gibco). MDA-MB-231, MDA-MB-436, MDA-MB-468, HCC-70, BT-20, BT-549, T47D, MDA-MB-361, MCF-7 and BT-474 were originally sourced from the American Type Culture Collection (ATCC). All cell lines were grown in a Galaxy+incubator (RS Biotech) at 37°C with 5% CO_2_.

To generate RUNX1 overexpressing cells, hMEC-TERT were transfected with pBABE-Puro-*RUNX1* or pBABE-Puro (kindly provided by Dr Anna Kilbey) through electroporation using Nucleofector Kit V, program T-013 (Amaxa, Lonza). After electroporation, cells were allowed to recover for 24 h and then selected in puromycin selection media (10 µg/ml) for 2 weeks.

### Western blot

Nuclear extracts were prepared from mammary cell lines using NE-PER Nuclear and Cytoplasmic Extraction Reagents (Thermo Scientific, Cat No 78833) as per kit instructions. Protein extracts were resolved on 10% NuPAGE Novex Bis-Tris gels (Life Technologies) and transferred to Hybond-ECL nitrocellulose membranes (Amersham). Membranes were probed with antibodies to RUNX1 (HPA004176, Sigma), HDAC2 (sc-6296, Santa Cruz) and GAPDH (Cell Signalling).

## Results

### Characterisation of RUNX1 expression in human breast cancer

**Figure 1:**
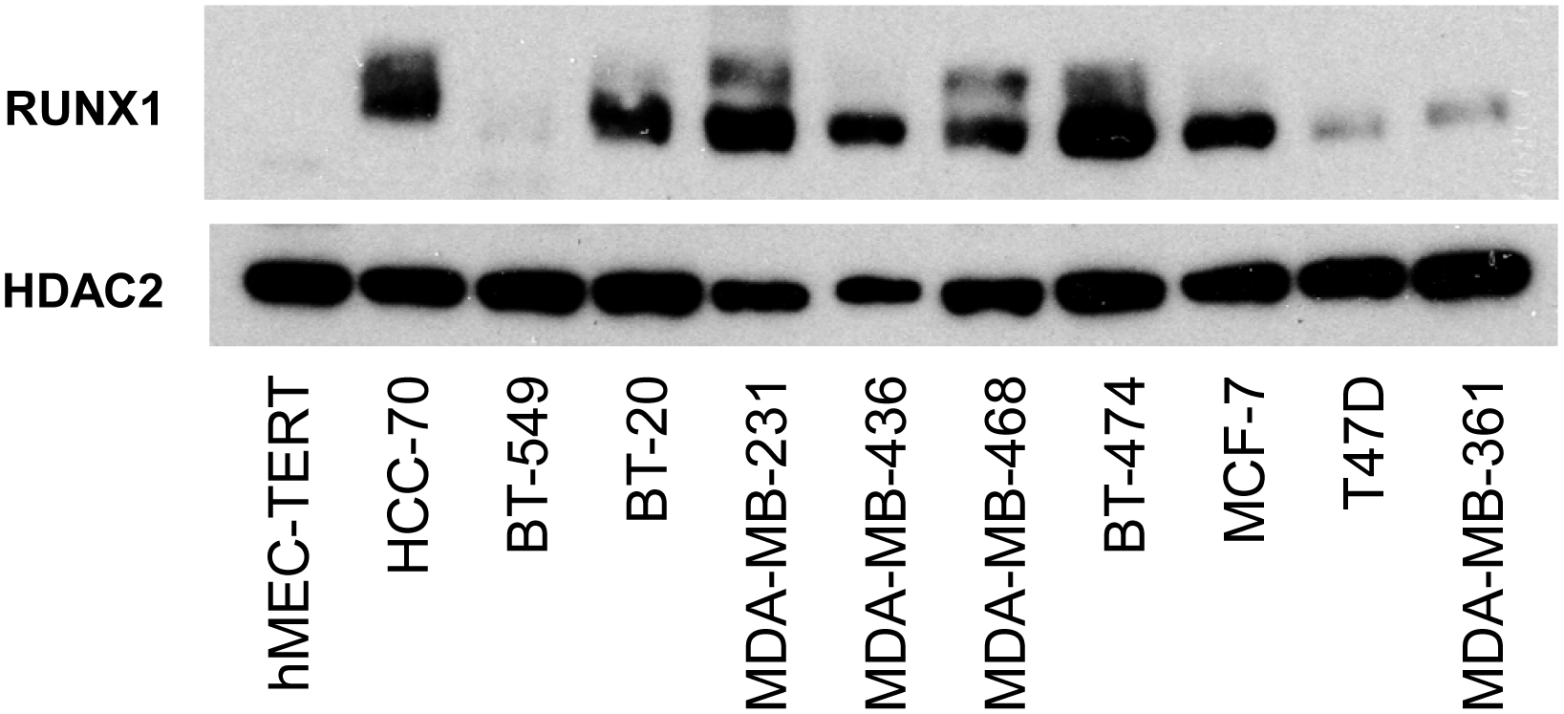
Expression of RUNX1 in human breast cancer cell lines. RUNX1 expression by western blot on a panel of human breast cancer cell lines with basal–like (HCC-70, BT-549, BT-20, MDA-MB-231, MDA-MB-436, MDA-MB-468) and luminal-like (BT-474, MCF-7, T47D, MDA-MB-361) features. HDAC2 used as a loading control. hMEC-TERT; immortalized human mammary epithelial cells.

RUNX1 expression was first tested on a panel of breast cancer cell lines. The chosen cell lines included normal human mammary epithelial cells derived from primary tissue and immortalised with TERT expression (hMEC-TERT), 6 basal-like (HCC-70, BT-549, BT-20, MDA-MB-231, MDA-MB-436, MDA-MB-468) and 4 luminal-like (BT-474, MCF-7, T47D, MDA-MB-361) breast cancer cell lines. Significantly, RUNX1 expression was not detectable in normal hMEC-TERT but was expressed in all breast cancer cell lines tested with the exception of BT-549 (Figure 1). These results suggest that RUNX1 expression could be dysregulated in human breast cancer.

**Figure 2:**
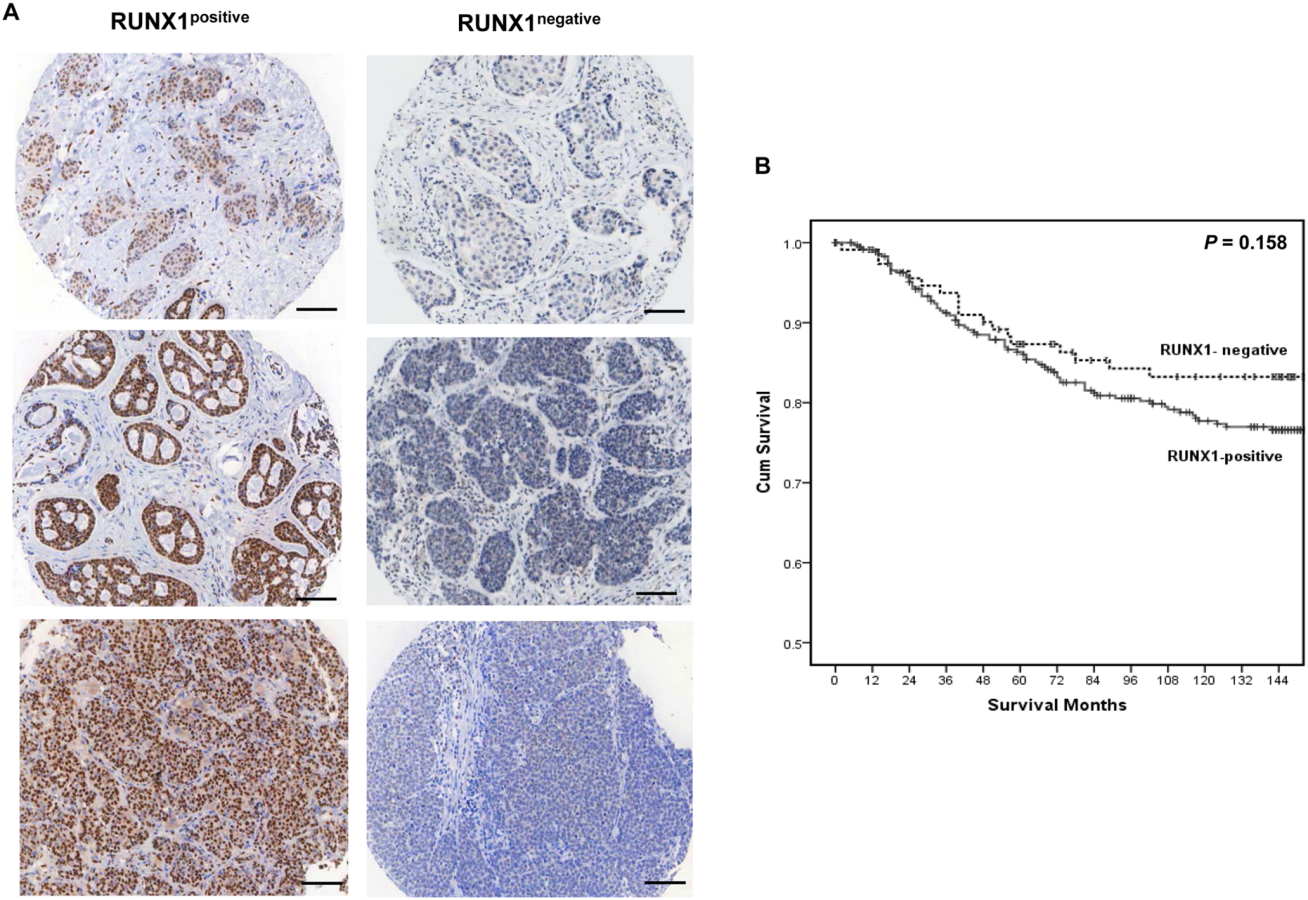
RUNX1 expression and cancer-specific survival in primary operable breast cancer. (A) Representative examples of invasive breast carcinomas in a tissue microarray containing 483 breast cancers which were positive (left) and negative (right) for RUNX1 expression. Note the nuclear staining in the tumour epithelium. Scale bar represents 100 µm. (B) The association between the absence and the presence of RUNX1 and cancer-specific survival in primary operable breast cancer (n = 483). Survival curves are plotted for patients with cancers scored positive for RUNX1 (solid line), or negative for RUNX1 expression (dotted line). P>0.1, P-value calculated using Log Rank (Mantel-Cox) test.

To investigate if RUNX1 expression influenced clinical outcome in primary breast tumours, a tissue microarray (TMA) containing biopsies from 483 patients with operable invasive ductal breast cancer [18] was stained for RUNX1. Baseline clinico-pathological characteristics of the patients included in the TMA are shown in Table 1. The invasive cancers showed different degrees of RUNX1 expression, predominantly localized to the nucleus (Figure 2A). RUNX1 expression in the tumour epithelium was determined by histoscore which takes into account the percentage of positive signal and staining intensity. Patients were divided into two groups: RUNX1 negative (histoscore = 0, n = 117) and RUNX1 positive (histoscore >0, n = 366). The relationships between RUNX1 expression and clinico-pathological characteristics in patients with primary operable ductal invasive breast cancer are shown in Table 2. In the whole cohort a number of factors were identified to be associated with positive RUNX1 protein levels including age (P<0.05), ER status (P<0.10), PR status (P<0.05), tumour lymphocyte and macrophage infiltrate (all P<0.05). In contrast, in those patients with triple negative receptor status only the tumour CD4 lymphocytic infiltrate was significantly associated with positive RUNX1 protein levels (Table 3; P<0.05).

**Table 1:** Clinico-pathological characteristics of patients with primary operable invasive ductal breast cancer.

Clinico-pathological characteristics (total)	Patients (n%)
**Age** (≤50/>50 years) (n = 483)	141 (29%)/342 (71%)
**Size** (≤20/21–50/>50 mm) (n = 481)	280 (58%)/186 (39%)/15 (3%)
**Tumour type** (Special type/lobular/ductal) (n = 483)	23 (5%)/33 (7%)/427 (88%)
**Grade** (I/II/III) (n = 481)	88 (18%)/202 (42%)/191 (40%)
**Involved lymph node** (Negative/positive) (n = 478)	268 (56%)/210 (44%)
**Oestrogen -receptor status** (ER−/ER+) (n = 481)	184 (38%)/297 (62%)
**Progesterone -receptor status** (PR−/PR+) (n = 480)	266 (55%)/214 (45%)
**HER2 status** (HER2−/HER2+) (n = 466)	393 (84%)/73 (16%)
**Lymphovascular invasion** (Absent/present) (n = 372)	198 (53%)/174 (47%)
**Microvessel density** (CD34+) (Low/medium/high) (n = 450)	157 (35%)/150 (33%)/143 (32%)
**Ki-67 status** (Low Ki-67/high Ki-67) (n = 468)	353 (75%)/115 (25%)
**Tumour necrosis** (Absent/present) (n = 473)	213 (45%)/260 (55%)
**TUNEL** (Low/high) (n = 417)	235 (56%)/182 (44%)
**General inflammatory infiltrate** (Low high) (n = 473)	334 (71%)/139 (29%)
**Tumour CD4**+**T- lymphocytic infiltrate** (Low/medium/high) (n = 474)	217 (46%)/93 (20%)/164 (34%)
**Tumour CD8**+**T- lymphocytic infiltrate** (Low/medium/high) (n = 474)	162 (34%)/154 (32.5%)/158 (33%)
**Tumour CD138**+**B- lymphocytic infiltrate** (Low/medium/high) (n = 473)	265 (56%)/60 (13%)/148 (31%)
**Tumour CD68**+**macrophages infiltrate** (Low/medium/high) (n = 471)	141 (30%)/164 (35%)/166 (35%)
**Loco-regional treatment** (Lumpectomy+radiotherapy/mastectomy+radiotherapy) (n = 483)	170 (35%)/313 (65%)
**Systemic treatment** (ER-based treatment) (hormonal/hormonal+chemotherapy/chemotherapy/none) (n = 476)	252 (53%)/98 (20%)/103 (22%)/23 (5%)
**RUNX1 (Negative/positive) (n = 483)**	117 (24%)/366 (76%)

(n = 483).

**Table 2:** The relationship between RUNX1 and clinico-pathological characteristics of patients with primary operable invasive ductal breast cancer.

Clinico-pathological characteristics (total)	RUNX1 Negative(n = 117)	RUNX1 Positive(n = 366)	p-value
**Age** (≤50/>50 years) (n = 483)	25/92	116/250	**0.033**
**Size** (≤20/21–50/>50 mm) (n = 481)	70/43/4	210/143/11	0.769
**Tumour type** (Special type/lobular/ductal) (n = 483)	8/9/100	15/24/327	0.197
**Grade** (I/II/III) (n = 481)	17/57/43	71/145/148	0.891
**Involved lymph node** (Negative/positive) (n = 478)	70/46	198/164	0.287
**Oestrogen -receptor status** (ER−/ER+) (n = 481)	53/64	131/233	0.072
**Progesterone -receptor status** (PR−/PR+) (n = 480)	75/42	191/172	**0.03**
**HER2 status** (HER2−/HER2+) (n = 466)	97/16	296/57	0.613
**Lymphovascular invasion** (Absent/present) (n = 372)	48/36	150/138	0.414
**Microvessel density** (CD34+) (Low/medium/high) (n = 450)	44/31/30	113/119/113	0.143
**Ki-67 status** (Low Ki-67/high Ki-67) (n = 468)	87/26	266/89	0.658
**Tumour necrosis** (Absent/present) (n = 473)	52/63	161/197	0.963
**TUNEL** (Low/high) (n = 417)	51/42	184/140	0.738
**General inflammatory infiltrate** (Low high) (n = 473)	81/34	253/105	0.962
**Tumour CD4**+**T- lymphocytic infiltrate** (Low/medium/high) (n = 474)	63/22/30	154/71/134	**0.015**
**Tumour CD8**+**T- lymphocytic infiltrate** (Low/medium/high) (n = 474)	55/28/32	107/126/126	**0.004**
**Tumour CD138**+**B- lymphocytic infiltrate** (Low/medium/high) (n = 473)	80/11/24	185/49/124	**0.001**
**Tumour CD68**+**macrophages infiltrate** (Low/medium/high) (n = 471)	50/39/24	91/125/142	**<0.001**
**Loco-regional treatment** (Lumpectomy+radiotherapy/mastectomy+radiotherapy) (n = 483)	39/78	131/235	0.628
**Systemic treatment** (ER-based treatment) (hormonal/hormonal+chemotherapy/chemotherapy/none) (n = 476)	65/23/21/6	187/75/82/17	0.42
**Cancer specific survival** (months)*	156 (146–165)	149 (143–155)	0.158

*Mean (95%CI).

**Table 3:** The relationship between RUNX1 and clinico-pathological characteristics of patients with triple negative primary operable invasive ductal breast cancer.

Clinico-pathological characteristics (total)	RUNX1 Negative	RUNX1 Positive	p-value
**Age** (≤50/>50 years) (n = 483)	10/22	33/53	0.477
**Size** (≤20/21–50/>50 mm) (n = 481)	17/15/0	43/38/4	0.537
**Tumour type** (Special type/lobular/ductal) (n = 483)	5/0/27	6/2/78	0.223
**Grade** (I/II/III) (n = 481)	0/9/23	4/14/66	0.857
**Involved lymph node** (Negative/positive) (n = 478)	20/12	48/38	0.515
**Lymphovascular invasion** (Absent/present) (n = 372)	16/9	37/35	0.278
**Microvessel density** (CD34+) (Low/medium/high) (n = 450)	8/11/11	30/17/37	0.928
**Ki-67 status** (Low Ki-67/high Ki-67) (n = 468)	22/9	69/16	0.239
**Tumour necrosis** (Absent/present) (n = 473)	7/25	16/70	0.691
**TUNEL** (Low/high) (n = 417)	14/8	48/17	0.363
**General inflammatory infiltrate** (Low high) (n = 473)	10/22	40/46	0.137
**Tumour CD4**+**T- lymphocytic infiltrate** (Low/medium/high) (n = 474)	18/3/10	25/17/42	**0.016**
**Tumour CD8**+**T- lymphocytic infiltrate** (Low/medium/high) (n = 474)	11/7/13	28/16/40	0.675
**Tumour CD138**+**B- lymphocytic infiltrate** (Low/medium/high) (n = 473)	19/3/9	42/8/34	0.251
**Tumour CD68**+**macrophages infiltrate**(Low/medium/high) (n = 471)	14/7/10	36/17/31	0.71
**Loco-regional treatment** (Lumpectomy+radiotherapy/mastectomy+radiotherapy) (n = 483)	16/16	35/51	0.367
**Systemic treatment** (ER-based treatment) (hormonal/hormonal+chemotherapy/chemotherapy/none) (n = 476)	8/7/14/2	21/8/46/9	0.356
**Cancer specific survival** (months)*	163 (148–179)	129 (114–144)	**0.013**

*Mean (95%CI).

(n = 118).

The relationship between RUNX1 expression and clinical outcome was then assessed by looking at cancer-specific survival in the full cohort as shown in Figure 2B. Survival analyses showed no significant difference between RUNX1 negative (mean of 156 months - 95% confidence interval, 146–165 months) and RUNX1 positive tumours (mean of 149 months - 95% confidence interval, 143–155 months) (Figure 2B). Minimum follow-up was 142 months; the median follow-up of the survivors was 164 months. 110 patients developed recurrence; 18 local, 71 distant, 6 with both and 15 with no information available. During the follow up period, 207 patients died and of these, 95 deaths could be directly attributed to their disease.

### Impact of RUNX1 expression on survival in breast cancer according to hormonal status

**Figure 3:**
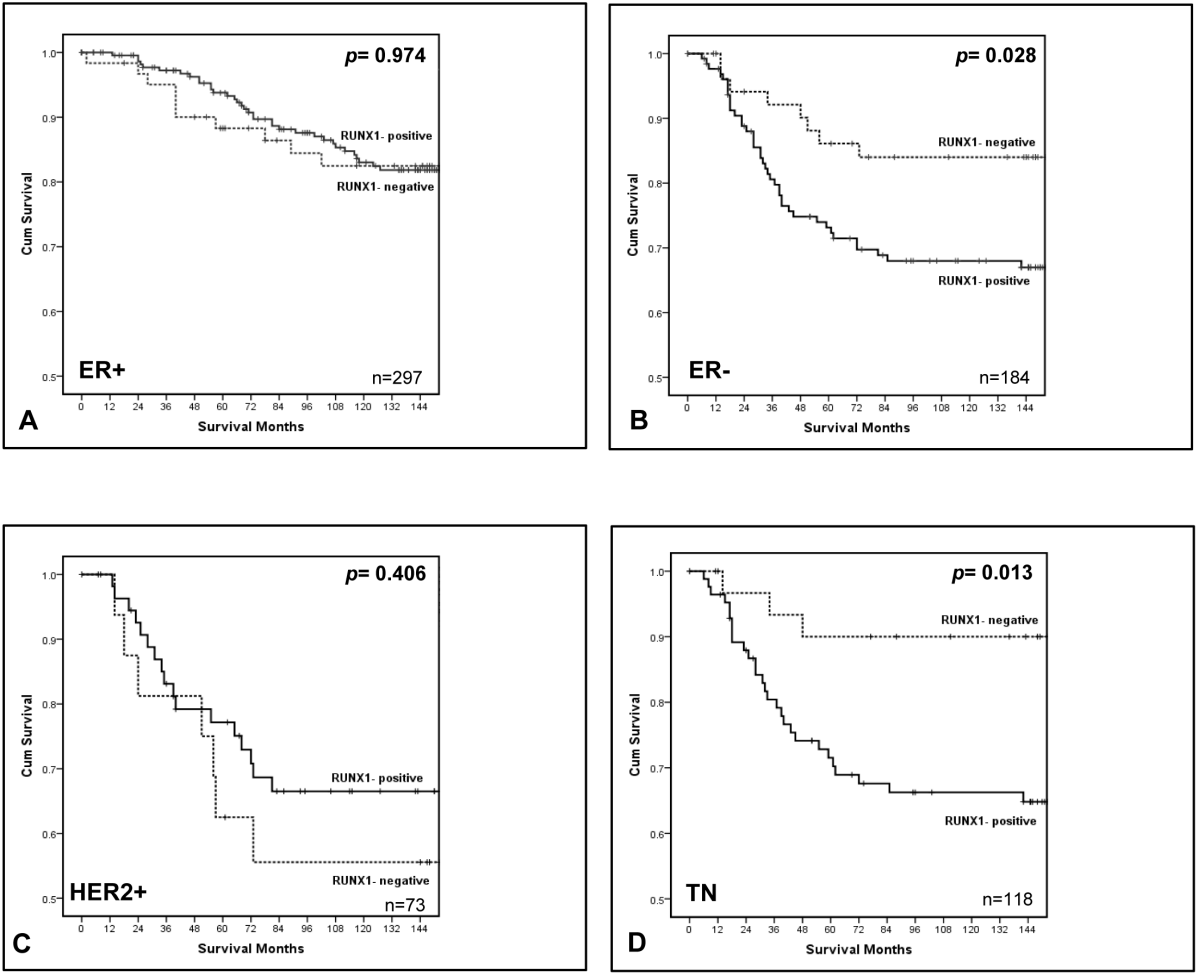
RUNX1 expression and cancer-specific survival in different subtypes of breast cancer. The association between the absence and the presence of RUNX1 and cancer-specific survival in patients with (A) ER-positive, (B) ER-negative, (C) HER2-positive and (D) triple negative (TN) primary operable breast cancer. Survival is plotted for patients with cancers positive for RUNX1 (solid line), or those with no RUNX1 expression (dotted line). ER+ cohort (n = 297), p = 0.974; ER– (n = 184), p = 0.028; HER2+ cohort (n = 73), p = 0.406; TN cohort (n = 118), p = 0.013. P-values calculated using Log Rank (Mantel-Cox) test.

To define the prognostic impact of RUNX1 expression in different breast cancer subtypes, the patient cohort was divided into 4 subgroups accordingly to their receptor status (ER+, ER–, HER2+ and ER−/PR−/HER2−). The distribution of RUNX1 positive and negative samples in relation to hormonal status (ER/PR/HER2) of the full cohort is shown in Table S1. No specific enrichment of RUNX1 was detected in any one of the hormonally defined subgroups, similar to what has been shown at a transcriptomic level [26]. The relationship between RUNX1 expression and clinical outcome was then assessed by looking at cancer-specific survival in each breast cancer subtype. Survival analyses showed no difference between the RUNX1 positive and negative groups in the ER+ and HER2+ patients (Figure 3A, 3C). However, RUNX1 showed a positive association with worse prognosis in the ER− (Figure 3B) and in the triple negative (TN) (Figure 3D) patients. In the TN subgroup mean cancer-specific survival of RUNX1 positive patients was 129 months (95% CI, 114–144 months) compared to 163 months (95% CI, 148–179 months) of the RUNX1 negative group. The relationships between RUNX1 and clinico-pathological characteristics were examined in patients with ER– (Table S2) and TN tumours (Table 3). In addition to a significant increase in CD4+T-lymphocytic infiltrate (P<0.05), RUNX1 positive tumours showed a significant increase in CD138+B- lymphocytic infiltrate (P<0.05) in ER- patients (Table S2). In a univariate analysis the presence of RUNX1 was associated with poorer cancer-specific survival for patients with ER- tumours (Table 4, *p*<0.05) and showed a tendency towards significance as an independent prognostic marker in multivariate analysis (p = 0.058). More interestingly, RUNX1 was significantly associated with poorer recurrence-free survival and cancer-specific survival for patients with triple negative disease (Table 5, p = 0.046 and p = 0.022 respectively). Using multivariate analysis RUNX1 expression was an independent prognostic marker for cancer specific-survival in the TN subtype when assessed against established pathological prognostic factors such as tumour size, grade, tumour type and lymph node status (Table 5).

**Table 4:** The relationship between clinic-pathological characteristics of patients with ER- negative primary operable invasive ductal breast cancer and recurrence-free/cancer- specific survival.

	Recurrence-free survival				Cancer-specific survival			
	Univariatesurvival analysis		Multivariatesurvival analysis		Univariatesurvival analysis		Multivariatesurvival analysis	
Clinico-pathologicalcharacteristics (total)	Hazard ratio(95% CI)	*P*-value	Hazard ratio(95% CI)	*P*-value	Hazard ratio(95% CI)	*P*-value	Hazard ratio(95% CI)	*P*-value
**Age** (≤50/>50 years)	0.79 (0.45–1.38)	0.401			1.02 (0.57–1.83)	0.948		
**Size** (≤20/21–50/>50 mm)	2.01 (1.20–3.38)	0.008	1.98 (1.18–3.33)	0.01	2.02 (1.20–3.40)	0.008	1.81 (1.04–3.13)	0.035
**Tumour type** (Special type/lobular/ductal)	2.26 (0.81–6.36)	0.121			3.48 (0.78–15.56)	0.103		
**Grade** (I/II/III)	1.10 (0.69–1.74)	0.701			1.71 (0.94–3.11)	0.077		0.435
**Involved lymph node** (Negative/positive)	1.91 (1.07–3.39)	0.027		0.108	2.83 (1.54–5.21)	0.001	2.24 (1.19–4.24)	0.013
**RUNX1** (Negative/positive)	1.77 (0.88–3.54)	0.108			2.29 (1.07–4.88)	0.033	2.09 (0.97–4.48)	0.058

**Table 5:** The relationship between clinic-pathological characteristics of patients with triple negative primary operable invasive ductal breast cancer and recurrence-free/cancer- specific survival.

	Recurrence-free survival				Cancer-specific survival			
	Univariatesurvival analysis		Multivariatesurvival analysis		Univariatesurvival analysis		Multivariatesurvival analysis	
Clinico-pathologicalcharacteristics (total)	Hazard ratio(95% CI)	*P*-value	Hazard ratio(95% CI)	*P*-value	Hazard ratio(95% CI)	*P*-value	Hazard ratio(95% CI)	*P*-value
**Age** (≤50/>50 years)	0.95 (0.44–2.04)	0.888			1.18 (0.57–2.46)	0.659		
**Size** (≤20/21–50/>50 mm)	2.53 (1.23–5.21)	0.012	2.31 (1.14–4.65)	0.019	2.76 (1.45–5.25)	0.002	2.63 (1.36–5.09)	0.004
**Tumour type** (Special type/lobular/ductal)	5.33 (0.36–78.77)	0.223			3.18 (0.73–13.83)	0.122		
**Grade** (I/II/III)	0.97 (0.49–1.93)	0.926			1.40 (0.64–3.07)	0.404		
**Involved lymph node** (Negative/positive)	2.48 (1.15–5.35)	0.021	2.19 (1.00–4.81)	0.05	4.15 (1.91–9.02)	<0.001	4.01 (1.83–8.81)	0.001
**RUNX1** (Negative/positive)	3.40 (1.02–11.28)	0.046	3.00 (0.90–10.05)	0.075	4.03 (1.23–13.27)	0.022	3.83 (1.16–12.67)	0.028

## Discussion

Recent studies have highlighted a novel link for RUNX1 with breast cancer [13]–[16] but to date no direct assessment of RUNX1 protein has been carried out. We have now addressed this need and show that 366/483 (76%) of invasive breast carcinomas in a tumour tissue microarray were positive for RUNX1 protein. Our analysis reveals that there was no difference in overall survival of the full patient cohort, or in ER+, PR+ and HER2+ subgroups, when stratified on RUNX1 expression. However on univariate analysis, positive RUNX1 expression was significantly associated with poorer cancer-specific survival in the ER− (P<0.05) and triple negative (ER−/PR−/HER2−) (P<0.05) groups of patients. There was also a trend towards significance on multivariate Cox regression analysis of cancer-specific survival in ER− breast cancer (P<0.10) which reached significance in triple negative breast cancer (TNBC) (P<0.05). TNBC, which accounts for 15% to 20% of breast cancers, is an aggressive disease, associated with a significantly higher probability of relapse and poorer overall survival when compared with other breast cancer subtypes [27]. The lack of identified molecular targets in the majority of TNBCs means that chemotherapy remains the treatment of choice for these patients and unfortunately early relapse after chemotherapy is common [28]. Hence there is an urgent need for identification of better prognostic markers and novel therapeutic targets for this subtype [3],[29]. Only a few markers have so far been identified as having a predictive role for the prognosis of TNBC patients [30],[31]. Our results now suggest the utility of RUNX1 as a novel biomarker. In fact, regression analysis using the Cox’s proportional Hazards model confirmed that RUNX1 has prognostic value together with tumour size and lymph node status in the TNBC subgroup. Furthermore, multivariate analysis indicated that RUNX1 expression was independent of the established pathological prognostic factors currently used in the clinic making it a new putative prognostic indicator for TN tumours.

It is intriguing that even though RUNX1 was expressed in most breast cancer cell lines (Figure 1) and the majority of patients (Table S1) regardless of hormonal status, it was only in the hormone-negative patients that RUNX1 expression correlated with patient outcome. Our data therefore indicate that TNBCs expressing RUNX1 represent a group of tumours with the poorest prognosis and suggest that in this subtype RUNX1 may be contributing to tumour progression. If RUNX1 has a pro-oncogenic role in TNBCs, the question arises as to why this effect is not observed in tumours expressing the oestrogen receptor. It is possible that this is being masked by the capacity of RUNX1 to attenuate or distort ER signalling [26]. In this scenario RUNX1 would be exerting opposing effects; dampening ER driven growth yet inducing latent tumour aggression. These results may explain why in a recent flurry of papers ascribing a tumour suppressor role for RUNX1 in breast cancer [12]–[16], mutations were found almost exclusively in ER+ cancers. Of course it will be important to definitively establish a pro-oncogenic role for RUNX1 in TNBC and understand why expression is maintained in the most aggressive subtype. Interestingly our data are supported by several transcriptomic studies that have identified *RUNX1* as a possible oncogene in TNBC [32]–[34]. In particular *RUNX1* is among a 264 gene signature which correlates with a poor prognosis in TNBC [33] whilst another study demonstrated an inverse correlation between RUNX1 expression and survival in the claudin low group of TNBCs [32]. *RUNX1* was also among the top 20% differentially expressed genes in two TN subtypes identified by cluster analysis, namely the ‘mesenchymal stem-like’ (MSL), and ‘luminal androgen receptor’ (LAR) subtypes [34]. The MSL subtype also displays low expression of claudins 3, 4, and 7, supporting a possible link between RUNX1 expression and the claudin-low subtype.

Inflammation has been shown to represent a critical component of tumour progression [35]. Of significance, RUNX1 expression correlates with the presence of lymphocytic CD4+ infiltrate in TNBC. RUNX1 is one of the key factors that drives various aspects of T-cell differentiation including regulation of cytokine production [36]. We could speculate that in TNBC highly positive for RUNX1, that RUNX1 would drive a transcriptional programme in breast cancer cells resulting in production and secretion of high levels of cytokines which would then lead to recruitment of lymphocytic cells at the tumour site. Further studies will clarify the significance of the correlation between RUNX1 and CD4 lymphocytes in TNBC.

The widespread expression of RUNX1 in TNBC also suggests new therapeutic avenues for the treatment of TNBCs; for example the development of small-molecule inhibitors which bind to CBFβ and inhibit RUNX1 activity opens the possibility of a RUNX1-specific targeted therapy [37] [38]. In addition, work from Tumbar’s laboratory has shown that RUNX1 overexpression leads to STAT3 activation and is necessary for skin and oral cancer growth [39]. STAT3 is involved in human breast cancer with high STAT3 levels correlating with poorer survival [40]. If further studies can establish if RUNX1 overexpression and STAT3 activation are conserved in human breast cancer, and in TNBC in particular, this could pave the way for new treatment options based on the use of STAT3 inhibitors. Taken together our results identify RUNX1 as a new biomarker in TNBC and are opening exciting possibilities for the development of novel targeted therapies for this subgroup.

## Supporting Information

Figure S1 Validation of the RUNX1 antibody. RUNX1 antibody specificity was confirmed by western blot using known positive (T6i, hMEC-RUNX1) and negative (3SS, hMEC-Puro) controls. GAPDH used as a loading control. T6i; leukaemia cell line overexpressing RUNX1. 3SS; leukaemia cell line deleted for RUNX1. hMEC-TERT (immortalized human mammary epithelial cells) transfected with RUNX1 (hMEC-*RUNX1*) or empty vector (hMEC-Puro).
(TIF)

Table S1 Distribution of RUNX1 expression in relation to breast cancer hormonal status.
(DOCX)

Table S2 The relationship between RUNX1 and clinico-pathological characteristics of ER- patients with primary operable invasive ductal breast cancer (n = 184).
(DOCX)
