# Supplementary figures and images for: Expression of RUNX1 Correlates with Poor Patient Prognosis in Triple Negative Breast Cancer
Source: PLoS One. 2014 Jun 26;9(6):e100759. doi: 10.1371/journal.pone.0100759 (PMC4072705; doi:10.1371/journal.pone.0100759)

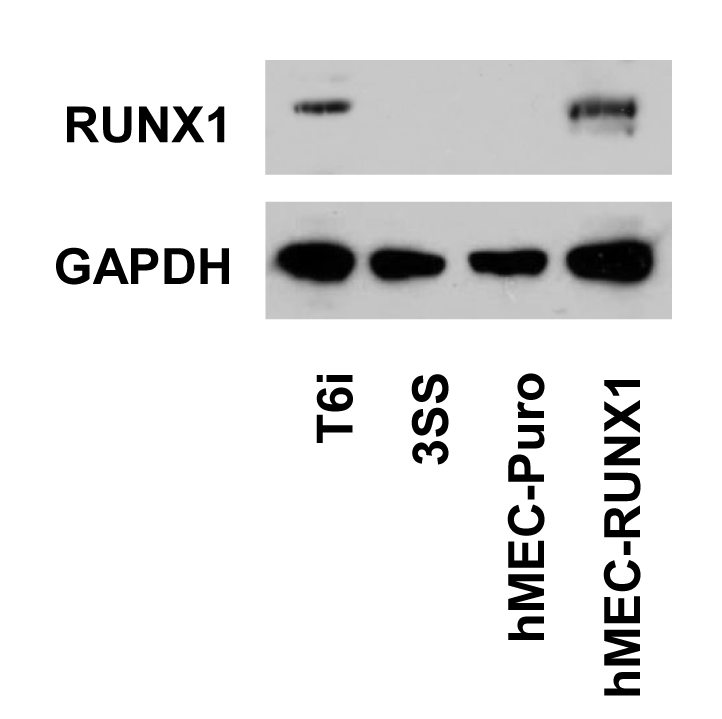

Supplement: Figure S1 — Validation of the RUNX1 antibody. RUNX1 antibody specificity was confirmed by western blot using known positive (T6i, hMEC-RUNX1) and negative (3SS, hMEC-Puro) controls. GAPDH used as a loading control. T6i; leukaemia cell line overexpressing RUNX1. 3SS; leukaemia cell line deleted for RUNX1. hMEC-TERT (immortalized human mammary epithelial cells) transfected with RUNX1 (hMEC-RUNX1) or empty vector (hMEC-Puro). (TIF) [file pone.0100759.s001.tif]
