# Supplementary material for: Expression of RUNX1 Correlates with Poor Patient Prognosis in Triple Negative Breast Cancer
Source: PLoS One. 2014 Jun 26;9(6):e100759. doi: 10.1371/journal.pone.0100759 (PMC4072705; doi:10.1371/journal.pone.0100759)
Supplement: Table S1 — Distribution of RUNX1 expression in relation to breast cancer hormonal status. (DOCX) [file pone.0100759.s002.docx]

| **Table S1. Distribution of RUNX1 expression in relation to breast cancer hormonal status** | | | | | | |
| --- | --- | --- | --- | --- | --- | --- |
| **Hormonal status** | **Total N** | **N %** | **RUNX1 +** | **% +** | **RUNX1 -** | **% -** |
| **Entire Cohort** |  |  |  |  |  |  |
|  | 483 |  | 366 | 75.8 | 117 | 24.2 |
| **ER** |  |  |  |  |  |  |
| Negative | 184 | 38.3 | 131 | 71.2 | 53 | 28.8 |
| Positive | 297 | 61.7 | 233 | 78.5 | 64 | 21.5 |
| **PR** |  |  |  |  |  |  |
| Negative | 266 | 55.4 | 191 | 71.8 | 75 | 28.2 |
| Positive | 214 | 44.6 | 172 | 80.4 | 42 | 19.6 |
| **HER2** |  |  |  |  |  |  |
| Negative | 393 | 84.3 | 296 | 75.3 | 97 | 24.7 |
| Positive | 73 | 15.7 | 57 | 78.1 | 16 | 21.9 |
| **TN** |  |  |  |  |  |  |
|  | 118 | 24.4 | 86 | 72.9 | 32 | 27.1 |
| Abbreviations: ER = Oestrogen receptor; PR = Progesterone receptor; HER2 = Human epidermal growth factor receptor 2; TN = Triple negative. Where information is not available on the full cohort (n=483), the number of patients with information is specified in brackets: ER (481), PR (480), HER2 (466). | | | | | | |
|  |  |  |  |  |  |  |
