# Supplementary material for: Expression of RUNX1 Correlates with Poor Patient Prognosis in Triple Negative Breast Cancer
Source: PLoS One. 2014 Jun 26;9(6):e100759. doi: 10.1371/journal.pone.0100759 (PMC4072705; doi:10.1371/journal.pone.0100759)
Supplement: Table S2 — The relationship between RUNX1 and clinico-pathological characteristics of ER- patients with primary operable invasive ductal breast cancer (n = 184). (DOCX) [file pone.0100759.s003.docx]

| **Table S2. The relationship between RUNX1 and clinico-pathological characteristics of ER- negative patients with primary operable invasive ductal breast cancer (n=184)** | | | |
| --- | --- | --- | --- |
| Clinico-pathological characteristics (total) | RUNX1 Negative (n=53) | RUNX1 Positive (n=131) | p-value |
|  |  |  |  |
| **Age** (≤50/ >50 years) | 14 / 39 | 51 / 80 | 0.109 |
| **Size** (≤20/ 21-50/ >50 mm) (n=183) | 28 / 25 / 0 | 63 / 61 / 6 | 0.327 |
| **Tumour type** (Special type/ lobular/ ductal) | 7 / 1 / 45 | 6 / 2 / 123 | 0.039 |
| **Grade** (I / II / III) (n=182) | 4 / 17 / 32 | 8 / 22 / 99 | 0.070 |
| **Involved lymph node** (Negative/ positive) (n=183) | 31 / 21 | 66 / 65 | 0.260 |
| **Progesterone -receptor status** (PR- / PR+) (n=183) | 49 / 4 | 123 / 7 | 0.578 |
| **HER-2 status** (HER-2 - / HER-2+) (n=172) | 36 / 13 | 90 / 33 | 0.968 |
| **Lymphovascular invasion** (Absent / present) (n=123) | 18 / 10 | 40 / 45 | 0.115 |
| **Microvessel density** (CD34+) (Low/ medium/ high) (n=169) | 14 / 17 / 17 | 39 /36/ 46 | 0.974 |
| **Ki-67 status** (Low Ki-67 / high Ki-67) (n=179) | 40 / 11 | 104 / 24 | 0.669 |
| **Tumour necrosis** (Absent/ present) | 15 / 38 | 28 / 103 | 0.316 |
| **TUNEL** (Low/ high) (n=140) | 23 / 15 | 72 / 30 | 0.259 |
| **General inflammatory infiltrate** (Low high) | 24 / 29 | 60 / 71 | 0.949 |
| **Tumour CD4+ T- lymphocytic infiltrate** (Low/ medium/ high) (n=177) | 28 /9/ 14 | 38 /23/65 | **0.001** |
| **Tumour CD8+ T- lymphocytic infiltrate** (Low/ medium/ high) (n=177) | 22 /13/ 16 | 39 /29/ 58 | 0.064 |
| **Tumour CD138+ B- lymphocytic infiltrate** (Low/ medium/ high) (n=176) | 34 /4/ 13 | 61 /11/ 53 | **0.027** |
| **Tumour CD68+ microphages infiltrate** (Low/ medium/ high) (n=176) | 27 /11/ 13 | 49 /28/ 48 | 0.069 |
| **Loco-regional treatment** (Lumpectomy + radiotherapy/ mastectomy + radiotherapy) | 19/34 | 45 / 86 | 0.847 |
| **Systemic treatment** (ER-based treatment) (hormonal/ hormonal + chemotherapy/ chemotherapy/ none) (n=180) | 17 /9/21/5 | 31 /20/66/ 11 | 0.273 |
| **Cancer specific survival** (months) **^*^** | 156 (141-170) | 132 (120-144) | **0.028** |

* Mean (95%CI)
